# Unveiling a Rare Pathology: Case Report of Carcinosarcoma of the Gallbladder

**DOI:** 10.1155/crip/2521664

**Published:** 2026-04-24

**Authors:** Gul Wymer, Laci-Rae Pitter, Susana Ferra

**Affiliations:** ^1^ Department of Pathology and Laboratory Medicine, HCA Florida Westside Hospital, Plantation, Florida, USA, lhsc.on.ca; ^2^ Nova Southeastern University Dr. Kiran C. Patel College of Allopathic Medicine, Davie, Florida, USA

**Keywords:** biliary tract cancer, case report, gallbladder carcinosarcoma, pathological diagnosis, poor prognosis, rare tumor

## Abstract

**Introduction and Importance:**

Gallbladder carcinosarcoma (GBCS) is a rare and aggressive biliary tract malignancy, constituting approximately 1.7% of gallbladder cancers, with fewer than 100 cases reported in the English literature as of 2023. These tumors, often affecting gynecological organs, feature both carcinomatous and sarcomatous components. They predominantly occur in females, with a mean presentation age of 66 years. Diagnosis is primarily based on pathological analysis, and the mainstay of treatment is surgical excision.

**Presentation of Case:**

We present a case of an 81‐year‐old female with no significant medical history, who was admitted after a fall. A CT scan revealed an 18.2 cm gallbladder mass extending into the liver and colon. The patient underwent tumor resection. Pathological examination confirmed a carcinosarcoma with osteoid and cartilage elements, supported by immunohistochemical staining as gallbladder primary.

**Clinical Discussion:**

Carcinosarcomas are composed of both epithelial (commonly adenocarcinoma) and mesenchymal (spindle cell) components. Their pathogenesis remains poorly understood. Typically diagnosed at advanced stages, these tumors have a poor prognosis, with survival rates ranging from 2.9 to 6 months. Gallbladder carcinosarcomas behave similarly to sarcomas, exhibiting rapid growth and resistance to both radiation and chemotherapy. Current treatment consensus involves surgical excision of the gallbladder, extrahepatic bile duct, regional lymphadenectomy, and possibly pancreaticoduodenectomy depending on tumor extent.

**Conclusion:**

Gallbladder carcinosarcoma is a rare and aggressive malignancy with a poor prognosis even following complete resection. Given the limited number of cases, further research is necessary to improve treatment strategies for these patients.

**Highlights**

• Gallbladder carcinosarcoma is an exceptionally rare biliary tract malignancy.
• Case presented with a massive tumor invading the liver and colon in an elderly female.
• Histology revealed adenocarcinoma with osteoid and cartilage sarcomatous elements.
• Add to scarce literature; prognosis remains poor despite complete surgical resection.

## 1. Introduction

Gallbladder carcinosarcoma represents a rare and aggressive biliary tract malignancy, accounting for approximately 1.7% of all gallbladder cancers [1]. A review of English literature up to 2023 identified fewer than 100 reported cases [1]. These tumors are characterized by the presence of both carcinomatous (epithelial) and sarcomatous (mesenchymal) components and have been observed to involve mainly the gynecological tract, less commonly otolaryngologic organs such as the thyroid gland and rarely reported in other organs [1]. The condition exhibits a female predominance (female: male ratio of approximately 2.7:1) with a mean age at presentation of 66 years [1, 2]. Patients often present with abdominal pain as the primary complaint, frequently accompanied by normal liver function tests (LFTs) and serum tumor marker levels [1, 2]. Typically diagnosed at advanced stages, gallbladder carcinosarcomas are associated with a poor prognosis, with reported survival ranging from 2.9 to 6 months [3]. Definitive diagnosis relies on pathological analysis, and surgical excision remains the mainstay of treatment [4]. This case report has been reported in line with the SCARE checklist [5].

## 2. Presentation of Case

**Figure 1:**
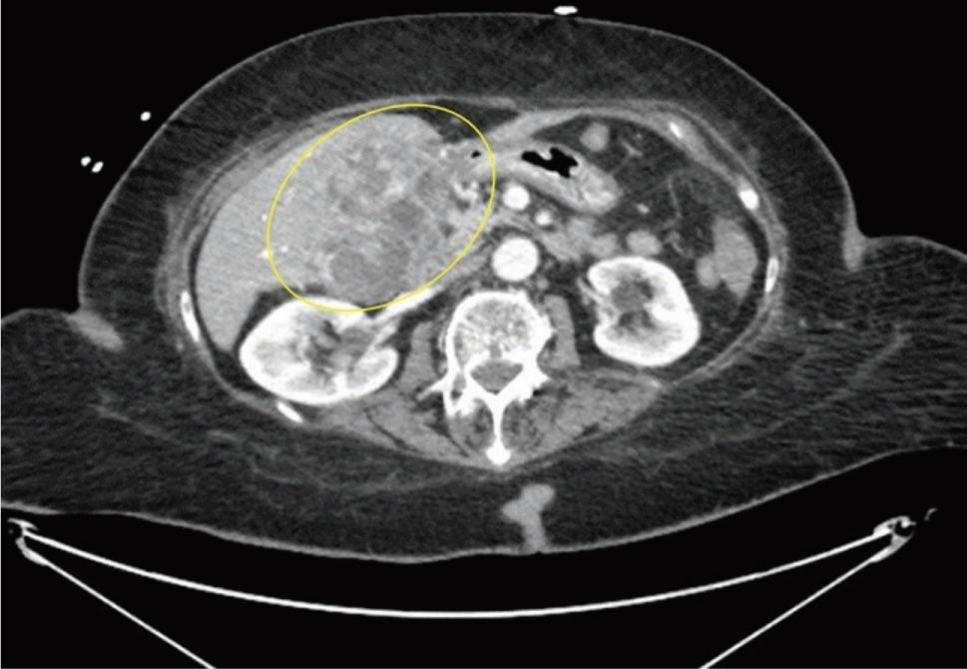
CT abdomen without contrast axial view shows a heterogeneous mass with calcifications 18.2 × 16.7 × 10.7 cm (yellow circle) centered in the gallbladder fossa, demonstrating extension into the liver and transverse colon.

An 81‐year‐old female with no known significant past medical history presented to the emergency department (ED) following a fall at home, reporting an inability to ambulate. The patient denied loss of consciousness or head trauma and reported a 1‐week history of generalized weakness, nonbloody emesis, and diarrhea. Upon initial evaluation, the patient was found to have severe sepsis and was admitted to the hospital. Further diagnostic workup included computed tomography (CT) imaging of the abdomen (Figure 1), which revealed a large (10.7 × 16.7 × 18.2 cm) heterogeneous mass with calcifications centered in the gallbladder fossa, demonstrating extension into the liver and transverse colon. Following biopsy confirmation of malignancy and exclusion of overt metastatic disease, the patient underwent surgical resection of the tumor.

**Figure 2:**
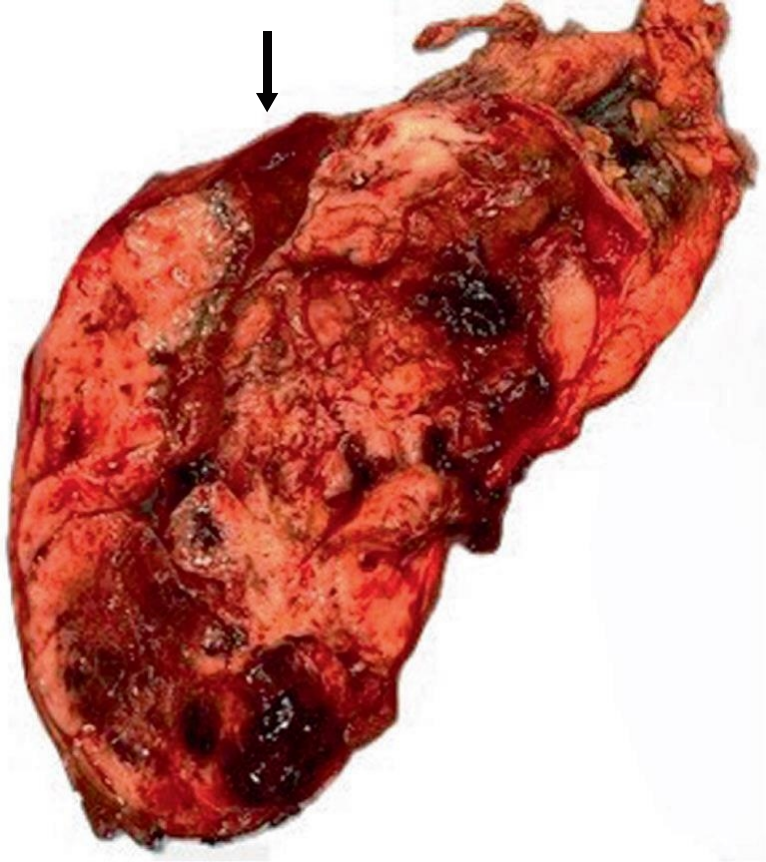
Gross photograph of the resected specimen. The lesion appears variegated multinodular light tan with areas of necrosis and hemorrhagic cysts. A rim of uninvolved liver tissue is seen in the superior aspect (arrow).

The en‐bloc resected specimen consisted of gallbladder mass, wedge of liver and segment of transverse colon attached to the gallbladder free wall. Sectioning revealed a 17.8 cm tan‐yellow multinodular necrotic and hemorrhagic solid and cystic tumor with calcified areas essentially replacing the entire gallbladder and with gross extension into the attached colon mucosa and adjacent liver (Figure 2).

Histopathologic examination of the biopsy specimen demonstrated in situ adenocarcinoma without evidence of invasive carcinoma or a sarcomatous component.

**Figure 3:**
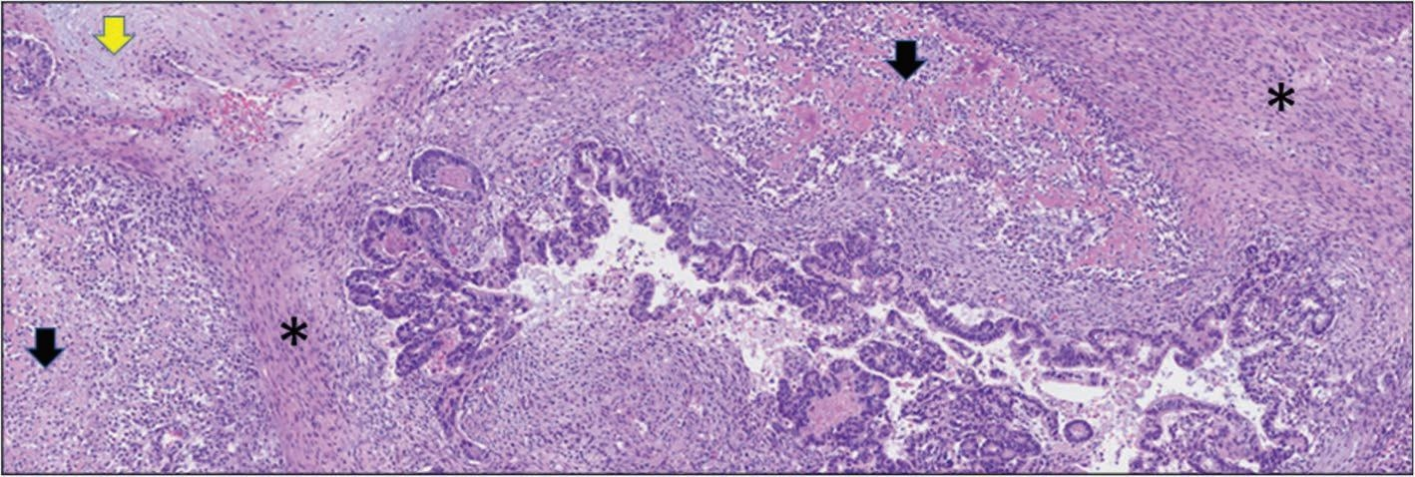
Photomicrographs of hematoxylin and eosin stained sections at 40× magnification demonstrating a variegated tumor with glandular (bottom center), spindle (asterisk), chondroid (yellow arrow), and osteoid (black arrow) components.

**Figure 4:**
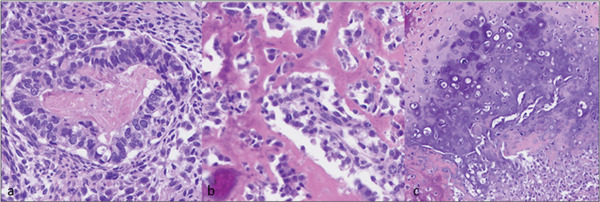
Photomicrographs of hematoxylin and eosin stained sections at 400× magnification demonstrating (a) adenocarcinoma gland, (b) osteoid formation, and (c) mature cartilage.

**Figure 5:**
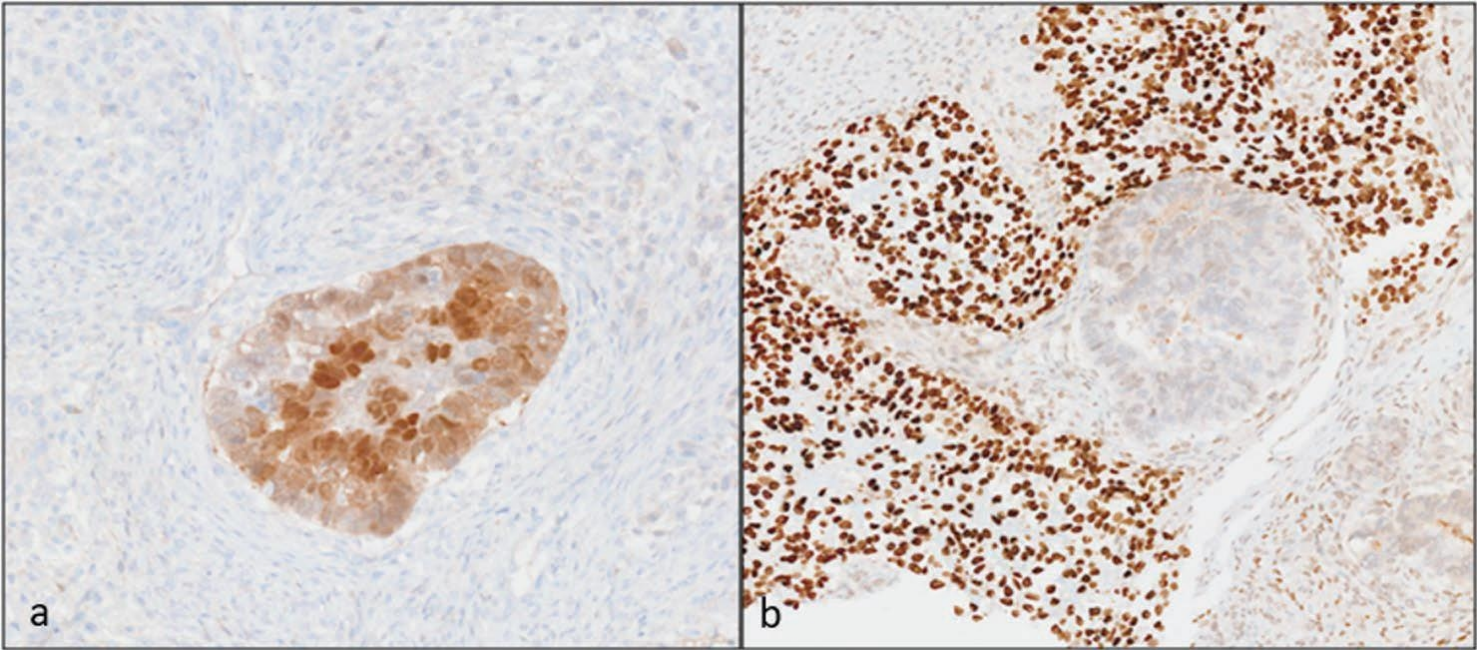
Photomicrographs of immunohistochemical stains at 200× magnification (a) CDX‐2 demonstrating weak positive staining on the adenocarcinoma component with negative spindle cell sarcoma in the background and (b) SATB2 negative adenocarcinoma gland at center with strong positive sarcoma in the background.

Light microscopy histologic examination reveals a variegated appearance with multiple tumor components (Figure 3). There was a predominant high‐grade adenocarcinoma component (Figure 4a, left) along with an in situ component in a background malignant spindle stroma with foci of frank osteoid formation (Figure 4b, center) and cartilage (Figure 4c, right) supporting a carcinosarcoma diagnosis. Negative PAX‐8 and ER (not shown), in addition to the absence of a GYN tract mass, excluded metastatic Mullerian origin. The carcinoma component was positive for CK7 and weak CDX‐2 (Figure 5a), whereas negative for CK20 and SATB2. The sarcoma was strongly positive for SATB2 (Figure 5b), confirming osteosarcoma lineage. The p53 was overexpressed in both components (not shown). MMR/MSI studies were stable.

A final diagnosis of gallbladder carcinosarcoma was rendered after histopathological evaluation. Despite achieving complete surgical excision, the patient was deemed ineligible for chemotherapy due to her age and comorbidities. By family decision, the patient was discharged to hospice care 10 days postoperatively and expired 8 days thereafter.

## 3. Discussion

Although gallbladder cancer is the most common tumor of the biliary tract, primary malignant tumors of the gallbladder are rare [3, 6]. The most common histological type is adenocarcinoma, comprising about 80%–95%, followed by anaplastic (2%–7%), squamous cell (1%–6%), adenosquamous (1%–4%), small cell (1%–3%) subtypes and carcinosarcoma (1.7%) [1, 3]. Carcinosarcomas are composed of epithelial components, adenocarcinoma (79.2%) and squamous cell carcinoma (9.4%), and mesenchymal components usually including undifferentiated spindle and stellate cells with varying heterogeneous elements such as chondrosarcoma, osteosarcoma, rhabdomyosarcoma, and leiomyosarcoma [3, 6]. The pathogenesis of this tumor is not fully understood; however, two main theories have been postulated. Multiclonal theory proposes the proliferation of two or more stem cells of separate epithelial and mesenchymal origin. The monoclonal theory suggests that sarcomatous and carcinomatous components are derived from a single pluripotent stem cell that subsequently develops atypical differentiation [4, 6].

GBCS has been reported to have two classic radiological features: the papillary growth into the lumen of gallbladder maintaining a smooth outer margin and speckled calcification within the tumor. However, because these findings are not seen in all cases of GBCS, preoperative diagnosis can be challenging. Pathological examination showing epithelial (carcinomatous) component positive for cytokeratin and mesenchymal (sarcomatous) component positive for vimentin is needed to make the precise diagnosis of GBCS [3].

In this case, the tumor consists of high‐grade adenocarcinoma glands in a background of malignant spindle stroma with foci of frank osteoid formation supporting the carcinosarcoma diagnosis. The sarcoma was strongly positive for SATB2, confirming osteosarcoma lineage.

Cases showing adenocarcinoma with osteosarcomatous differentiation appear to be particularly uncommon, and published reports emphasize that osteoid‐producing malignant mesenchymal areas may occur either as the dominant heterologous component or alongside additional chondroid elements [7, 8].

By comparison, rhabdomyosarcomatous differentiation in gallbladder carcinosarcoma has been documented in classic reports dating back several decades, highlighting skeletal muscle differentiation as another recognized, although rare, heterologous mesenchymal phenotype within this tumor spectrum [9–11].

Leiomyosarcomatous differentiation has also been described among the heterologous sarcomatous components of gallbladder carcinosarcoma; however, this finding should be distinguished from primary gallbladder leiomyosarcoma, a rare pure mesenchymal malignancy arising independently of epithelial carcinoma [11–13].

Overall, regardless of the specific mesenchymal subtype, gallbladder carcinosarcoma demonstrates highly aggressive biological behavior and is frequently diagnosed at an advanced stage. Complete surgical resection remains the only treatment associated with any meaningful survival benefit [1, 14].

Although carcinosarcomas are commonly reported in gynecological organs, negative PAX‐8 and lack of a GYN mass excluded the possibility of metastatic disease.

Patients often present with abdominal pain as the primary complaint, frequently accompanied by normal LFTs and serum tumor marker levels [1, 2]. A history of gallstones is commonly noted; however, a definitive association has not been established [3]. In contrast to other cases where patients usually have a history of gallstones or chronic cholecystitis, this patient had no known past medical history of gallbladder pathology and presented at a later age than the mean age of 66.

Gallbladder carcinosarcoma behaves biologically similar to sarcomas and are defined by rapid growth and resistance to both radiation and chemotherapy [1]. They can spread either hematogenously by lymphatic invasion or by direct invasion to adjacent organs and vessels [3]. Currently, the only option for curative treatment is surgical excision of the gallbladder and extrahepatic bile duct, regional lymphadenectomy, and possible pancreaticoduodenectomy depending on the extent of tumor invasion [1, 6]. To date, only one case of recurring GBCS with positive PD‐L1 expression has been recorded and it completely regressed after a combination of chemotherapy and immunotherapy [15].

## 4. Conclusion

Gallbladder carcinosarcoma is a rare, aggressive biliary tract malignancy with poor prognosis even after complete resection. Due to the small number of cases published, the etiology is poorly understood. This additional case serves to add to the pool of data in an effort to improve treatment options for patients.

## Author Contributions

Gul Wymer: writing paper, final approval. Laci‐Rae Pitter: writing paper, final approval. Susana Ferra: study design/concept, data analysis, writing paper, final approval, and guarantor.

## Funding

No funding was received for this manuscript.

## Disclosure

This research was supported (in whole or in part) by HCA Health‐care and/or HCA Healthcare‐affiliated entities. The views presented in this article represent those of the authors of the article and do not necessarily represent the official views of HCA Health‐care or any of its affiliated entities.

## Ethics Statement

Ethics approval of case reports or case series manuscripts is not required by the HCA Healthcare GME Institutional Review Board.

## Consent

Informed consent is waived based on our institution’s guidelines.

## Conflicts of Interest

The authors declare no conflicts of interest.

## Data Availability

Data are deidentified and released with permission.
